# Genistein as a potential inducer of the anti-atherogenic enzyme paraoxonase-1: studies in cultured hepatocytes *in vitro* and in rat liver *in vivo*

**DOI:** 10.1111/j.1582-4934.2012.01542.x

**Published:** 2012-09-26

**Authors:** Charlotte Schrader, Insa M A Ernst, Heike Sinnecker, Sebastian T Soukup, Sabine E Kulling, Gerald Rimbach

**Affiliations:** a Institute of Human Nutrition and Food Science Christian-Albrechts-University of KielKiel, Germany; b Department of Safety and Quality of Fruit and Vegetables, Max Rubner-Institut Federal Research Institute of Nutrition and FoodKarlsruhe, Germany

**Keywords:** aryl hydrocarbon receptor, oestrogen receptor, flavonoids, genistein, genistein metabolites, oxidized LDL, paraoxonase 1

## Abstract

A number of cardioprotective effects, including the reduced oxidation of the low-density lipoprotein (LDL) particles, have been attributed to dietary soy isoflavones. Paraoxonase 1 (PON1), an enzyme mainly synthesized in the liver, may exhibit anti-atherogenic activity by protecting LDL from oxidation. Thus, dietary and pharmacological inducers of PON1 may decrease cardiovascular disease risk. Using a luciferase reporter gene assay we screened different flavonoids for their ability to induce PON1 in Huh7 hepatocytes in culture. Genistein was the most potent flavonoid with regard to its PON1-inducing activity, followed by daidzein, luteolin, isorhamnetin and quercetin. Other flavonoids such as naringenin, cyanidin, malvidin and catechin showed only little or no PON1-inducing activity. Genistein-mediated PON1 transactivation was partly inhibited by the oestrogen-receptor antagonist fulvestrant as well as by the aryl hydrocarbon receptor antagonist 7-ketocholesterol. In contrast to genistein, the conjugated genistein metabolites genistein-7-glucuronide, genistein-7-sulfate and genistein-7,4′-disulfate were only weak inducers of PON1 transactivation. Accordingly, dietary genistein supplementation (2 g/kg diet over three weeks) in growing rats did not increase hepatic PON1 mRNA and protein levels as well as plasma PON1 activity. Thus, genistein may be a PON1 inducer in cultured hepatocytes *in vitro*, but not in rats *in vivo*.

## Introduction

Cardiovascular disease (CVD) is the principal cause of death in the Western hemisphere [1–3]. In contrast, incidence of CVD is relatively low in Asian populations [2, 4], which may be partly attributed to differences in diet [2, 5]. The traditional Asian diet is rich in soy which contains high amounts of isoflavones such as genistein and daidzein [2, 6–10]. Isoflavone uptake is low in Western as compared to Asian countries [2, 6, 11]. Thus, it has been hypothesized that soy isoflavones may exhibit beneficial cardiovascular effects [2]. A number of anti-atherogenic properties have been attributed to dietary isoflavones, including diminished low-density lipoprotein (LDL) oxidation [12–14]. Interestingly around 20% of blood isoflavones are present in the LDL fraction [12].

An elevated plasma concentration of LDL is a risk factor for CVD [15] as oxidation of LDL is regarded as a critical event in atherogenesis [16–18], the process leading to CVD. Oxidized LDL is taken up by macrophages *via* scavenger receptors which leads to the extensive accumulation of cholesterol in the intima media and results in the formation of foam cells [15, 19, 20]. The enzyme Paraoxonase-1; (PON1; EC 3.1.1.2/3.1.8.1) exhibits anti-atherogenic activity by protecting LDL from oxidation [21, 22]. To the best of our knowledge the exact underlying mechanism by which high-density lipoprotein (HDL)-associated PON1 decreases LDL oxidation is largely unknown. However, an interaction between HDL and LDL particles is possible, for example under participation of several transfer proteins such as cholesterol ester transfer protein [23] or phospholipid transfer protein [24]. Furthermore, it has been shown that PON1 hydrolyses lipid peroxides and cholesteryl linoleate hydroperoxides and hydroxides [21, 25–27]. Moreover, it has been suggested that PON1, apoliprotein A1 and lecithin:cholesterol acyltransferase display additive effects as far as the protection of LDL from oxidation is concerned [28]. PON1 is a high-density HDL associated serum enzyme which is mainly synthesized in the liver [29]. It has been shown that PON1-deficient mice are highly susceptible toward atherosclerosis [30]. However, the overexpression of PON1 may counteract atherogenesis and promote plaque stability in mice [31]. Both dietary and pharmacological induction of PON1 may play an important role in the prevention of CVD [32]. Dietary flavonoids may improve PON1 status, thereby mediating anti-atherogenic properties [33, 34]. Systematic studies regarding structure related activity of flavonoids in terms of their PON1-inducing activity are currently missing. Therefore, in this study we investigated the impact of flavonols, flavones, flavan-3-ols, flavanones, anthocyanidines and isoflavones on PON1 transactivation in cultured hepatocytes. The most potent flavonoid test component based on the cell culture data *in vitro* was the isoflavone genistein, which was then studied with regard to its effect on PON1 mRNA, protein and activity levels in rats *in vivo*.

## Materials & methods

### Chemicals

**Fig 1:**
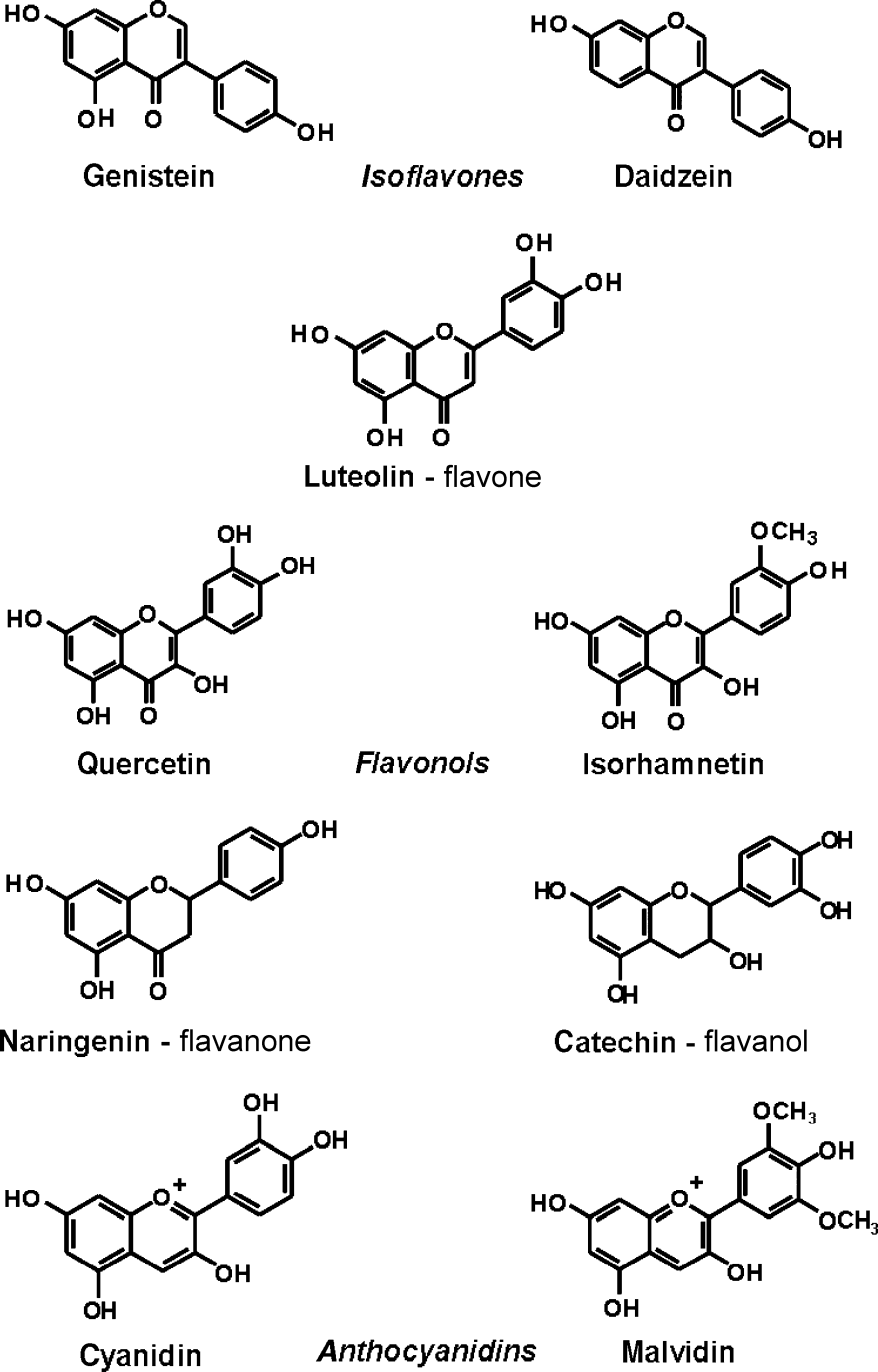
Chemical structures of the test compounds as used in the PON1 reporter gene assay in stably transfected Huh7 cells.

Resveratrol, daidzein, luteolin, quercetin, naringenin, (+)catechin hydrate and fulvestrant were purchased from Sigma-Aldrich Chemie GmbH (Steinheim, Germany). Isorhamnetin, cyanidin chloride and malvidin chloride were purchased from Extrasynthèse (Genay, France). Genistein was purchased from LC Laboratories (Woburn, MA, USA). The chemical structures of the test components are shown in Figure 1. The genistein metabolites genistein-7-glucuronide (G7-MGluc) and genistein-4′-glucuronide (G4′-MGluc) were purchased from Toronto Research Chemicals (North York, ON, Canada). Dihydrogenistein was purchased from APIN Chemicals LTD (Abingdon, UK). 6′-Hydroxy-*O*-desmethylangolensin (6′-OH-ODMA) was from Plantech UK (Reading, UK). [2,3,4^-13^C_3_]G7-MGluc and [3,4,8^-13^C_3_]daidzein were provided by Nigel Botting (University of St. Andrews, UK).

Genistein-7-sulfate (G7-MSulf) and genistein-7,4′-disulfate (G7,4′-DSulf) were synthesized according to the method of Fairley *et al*. with the following alterations [35]. A total quantity of 125 mg of genistein was dissolved in 5 ml dried pyridine, and the solution was cooled down on ice. 165 μl of chlorosulfonic acid was carefully added and the resulting solution was stirred overnight at room temperature. The solution was evaporated to dryness and the residue was resolved in 20% aqueous methanol containing 0.05 g/ml NaHCO_3_. The mixture of genistein mono- and disulfate in this solution were cleaned up using preparative LC. The compounds were identified by MS and NMR. The purity of G4′-MSulf and G7,4′-DSulf were >97%.

### Cell culture

Cell culture experiments were carried out in cultured hepatocytes. Huh7 liver hepatoma cells of human origin stably transfected with a 1000 bp fragment of the human PON1 promotor (PON1-Huh7; kindly provided by X. Coumoul and R. Barouki, INSERM, Paris, France) were cultivated in Dulbecco's modified Eagle's medium with 10% foetal calf serum, 100 U/ml streptomycin and 100 mg/ml penicillin (all from PAA, Coelbe, Germany).

### Cytotoxicity measurement

Cytotoxicity was determined *via* the Neutral Red assay [36]. PON1-Huh7 cells were seeded in 24-well plates (Fisher Scientific, Schwerte, Germany) at a density of ∼80,000 cells/cm^2^, pre-cultured for 24 hrs and treated with 1–25 μmol/l of the different flavonoids for 48 hrs, respectively. In brief, the culture medium containing the test substances was replaced with fresh serum-containing medium including 50 μg/ml of Neutral Red (Carl Roth GmbH + Co.KG, Karlsruhe, Germany). After incubation for 3 hrs, the medium was removed and the cells were extracted using a solution comprising 50:49:1 (v/v/v) ethanol, water and glacial acetic acid. The absorbance was measured in a plate reader (Labsystems, Helsinki, Finland) at 540 nm.

### Reporter gene assay

PON1-Huh7 cells were seeded at an initial density of ∼80,000 cells/cm^2^ (24-well plates) and incubated with the highest non-toxic concentration of flavonoids and resveratrol, respectively, for 48 hrs as recently described [33]. In a second series of experiments, cells were incubated with medium supplemented with 50 μmol/l fulvestrant and 20 μmol/l 7-ketocholesterol, respectively, in the absence or presence of 25 μmol/l genistein for 48 hrs. Afterwards, the cells were washed with PBS, lysed and subjected to luciferase activity measurement (Luciferase assay system; Promega, Madison, WI, USA) by luminescence reading (Infinite 200 reader; Tecan, Crailsheim, Germany) and normalized to total cell protein.

### Animals and Diets

The animal experiment was performed in accordance with the German regulations for animal welfare and approved by the Ministry of the federal state of Schleswig-Holstein [MLUR, Kiel, Germany, No. V312-72241.121-33(88-7/09)]. Twelve male Wistar Unilever rats (HsdCpb:WU, Harlan and Winkelmann, Germany; initial body weight ∼90 g) were housed in pairs in macrolon cages in a controlled environment (21 ± 2°C and 55 ± 5% relative humidity, 12 hrs light-dark cycle). Animals were fed either a flavonoid-free, semi-synthetic diet (ssniff special diets GmbH, Germany) or the same diet enriched with genistein at a concentration of 2 g/kg [37]. This refers to a genistein concentration which exceeds genistein concentrations that can be achieved because of a genistein-rich diet in humans [38]. Thus, the genistein concentration as used in this pilot study should rather be considered as a pharmacological dosage. However, it should be considered that genistein is also available as dietary supplement containing substantial genistein concentrations [39]. Experimental diet (47.2% corn starch, 24% casein, 11% glucose, 5% glucose, 3.8% coconut oil concentrate, 2% corn oil, 6% mineral premix, 1% vitamin premix) and water were provided *ad libitum*.

The rats were randomly divided into two groups of six animals each and fed the experimental diets for 22 days. Food intake (19.4–22.9 g/animal) and final body weight (218–259 g) were not significantly different between both groups [37]. At the end of the experiment, the rats were food deprived for 12 hrs, anaesthetized with carbon dioxide and decapitated. Liver tissue was quickly excised, rinsed with 0.9% NaCl solution, frozen in liquid nitrogen and stored at −80°C until further analysis. Blood was collected in tubes containing lithium-heparin coated beads, centrifuged to generate plasma (3000 *g*, 10 min., 4°C) and stored at −80°C.

### RNA isolation and real time qRT-PCR

Total RNA was isolated from rat liver tissue using RNeasy Mini kit (Qiagen, Hilden, Germany) and RNA was quantified photometrically (Spectrophotometer DU800; Beckman Coulter, Krefeld, Germany). RNA quality was controlled by gel electrophoresis. Real-time PCR was performed as one step procedure with QuantiTect SYBR Green RT-PCR kit (Qiagen). Quantitation was done using a standard curve. Primers were designed by standard tools (Spidey, Primer3, NCBI Blast) and purchased from MWG (Ebersberg, Germany). A 144 bp fragment of the rat PON1 gene (Gene ID 84024), specific for this isoform (no homology to PON2 and PON3 mRNA) was amplified using forward primer 5′–AAGCGGGTGCTGAAGACTTA–3′ and reverseprimer 5′–CTGCTGGCTCCTTCTCATTC-3′ and normalized to the mRNA levels of the housekeeping gene betaActin (Gene ID 81822; 165 bp fragment; primer forward 5′-GGGGTGTTGAAGGTCTCAAA-3′ and reverse primer 5′-TGTCACCAACTGGGACGATA-3′).

### Liver tissue preparation

Liver homogenates were prepared 1:10 with ice cold phosphate buffered saline (PBS, pH 7.4) and centrifuged at 3800 *g* at 4°C for 10 min. The supernatant was stored at –80°C until further use.

### Western blotting

Liver cell protein from rat tissue samples (30 mg) as well as Huh7 whole cell protein lysates were prepared using RIPA buffer (50 mmol/l Tris-HCl, 150 mmol/l NaCl, 0.5% deoxycholate, 0.1% SDS, and 1% NP-40; pH 7.4 with protease-inhibitor-cocktail, 1:100; Sigma-Aldrich, St. Louis, MO, USA) by incubation on ice for 30 min. and subsequent centrifugation at 12,000 *g* (4°C, 30 min.). Protein concentration was determined in the supernatants by the bicinchoninic acid assay (Pierce, IL, USA). 40 μg protein were separated on a 12% SDS/polyacrylamide gel and transferred onto an immunoblot polyvinylidenfluoride membrane. The membrane was blocked with 3% non-fat dried milk in Tris-buffered saline, pH 7.4, with 0.05% Tween-20 (TBS/T) for 2 hrs and probed with PON1-antibody (1:10,000; Abcam, Cambridge, UK), oestrogen receptor α (ERα) or β (ERβ) antibody (1:200; Santa Cruz Biotechnology, Heidelberg, Germany) and aryl hydrocarbon receptor (AhR) (1:200, Santa Cruz) respectively, at 4°C overnight. Then, the membranes were incubated with a goat anti-rabbit (1:4000; BioRad, Munich, Germany) or donkey anti-goat (1:3000; Santa Cruz) IgG secondary antibody conjugated with horseradish peroxidase for 45 min. Specific bands were visualized by enhanced chemiluminescence reagent on a ChemiDoc system and quantitated densitometrically using the program Quantity One® (all from BioRad). The membranes were stripped (strip buffer: 8 g glycine, 2.5 ml 37% HCl, 1l H_2_O) and subsequently incubated with rabbit polyclonal antibody against actin which was used as loading control (1:800; Santa Cruz) and proceeded as described above.

### Paraoxonase activity measurement

Activity of PON1 in plasma was measured *via* its arylesterase activity using phenylacetate as a substrate. Enzymatic activity was calculated from the molar absorbance coefficient of the produced phenol (1310/M/cm) with one unit of arylesterase activity defined as 1 μmol phenylacetate hydrolyzed/min. and expressed as kU/l serum. Blanks were simultaneously run to correct for spontaneous substrate breakdown. The rate of formation of phenol was determined using a working reagent consisting of 20 mM Tris/HCl buffer (pH 8.0), containing 4 mM phenylacetate and 1 mM CaCl_2_. Measurements were conducted at 270 nm and 25°C. Plasma PON1 activity was normalized to plasma HDL content.

### HDL-cholesterol measurement

Plasma HDL-cholesterol levels were determined using a commercially available spectrophotometric kit (Fluitest® HDL direct-kit; Analyticon® Biotechnologies AG, Lichtenfels, Germany).

### *In silico* promoter analysis

For promoter analysis, the PON1 gene was investigated by MatInspector Software http://www.genomatix.de to identify putative binding sites for oestrogen response elements and aryl hydrocarbon receptor-binding sites.

### Quantification of genistein and metabolites by LC/MS analysis

Rat plasma samples (200 μl) were thawed and 10 μl of each internal standard (1 μM [3,4,8-^13^C_3_]daidzein and 1 μM [2,3,4-^13^C_3_]G7-MGluc stock solution in DMSO) was added. The samples were acidified with 400 μl of 50 mM H_3_PO_4_ solution and purified *via* SPE extraction. Therefore, the SPE cartridges (Strata-X, 60 mg, 3 ml; Phenomenex, Torrance, CA, USA) were conditioned with 2 ml of methanol and equilibrated with 2 ml of 50 mM H_3_PO_4_ solution. After loading with the sample solutions the cartridges were washed with 2 ml of 10% (v/v) methanolic solution. The analytes were eluted with 2 ml of methanol and evaporated to dryness under a nitrogen stream. The residues were dissolved in 100 μl of 30% (v/v) methanolic solution and an aliquot of each sample was analysed by LC/MS as described below. For calibration, blank plasma was worked up as described above and analytes were added before analysis in an end concentration range between 2 and 1250 nM.

Rat liver samples (250 mg) were thawed and 10 μl internal standard (1 μM [3,4,8–^13^C_3_]daidzein and 1 μM [2,3,4-^13^C_3_]G7-MGluc stock solution in DMSO) was added. The samples were treated for 4 × 40 sec. with the FastPrep homogeniser using 1-mm silica spheres with intermediate cooling on ice (FastPrep-24; MP Biomedicals, Solon, OH, USA). The liver homogenates were acidified with 2 ml of 10% (w/v) citric acid solution and extracted two times by addition of 5 ml of ethyl acetate. The two ethyl acetate extracts resulting from each sample were combined and evaporated to dryness under a gentle nitrogen stream. The residues were dissolved in 100 μl of 30% (v/v) methanolic solution and an aliquot of each sample was analysed by LC/MS as described below. For calibration, blank liver was worked up as described above, and analytes were added before analysis in an end concentration range between 2 and 1250 nM.

The internal standard [3,4,8-^13^C_3_]daidzein was used to generate the calibration curves for genistein and dihydrogenistein and [2,3,4-^13^C_3_]G7-MGluc was used for 6′-OH-ODMA and both genistein-monoglucuronides. This decision was made on the basis of the average recovery rates of each analyte determined in plasma and liver tissue. The Supplemental Table 1 summarized the recovery rates. The modest recovery rate of 6′-OH-ODMA was acceptable because of the fact that the measured levels of this metabolite were very low, and therefore not of biological significance.

The LC-MS analyses were performed on an ABSciex QTrap 5500 mass spectrometer equipped with a Shimadzu LC system, which consisted of a controller (CBM-20A), a degasser (DGU-20A5), two pumps (LC-30AD), an autosampler (SIL-30AC) and a column oven (CTO-20AC). The LC-MS system was controlled by the software Analyst 1.5.2. The Turbo Spray ESI Source was operated in the negative mode. The source parameters were as follows: Curtain gas (CUR) 50 psi, ion spray voltage (IS) −4500 V, ion source gas-1 (GS 1) 80 psi, ion source gas-2 (GS 2) 70 psi, ion source gas-2 temperature (TEM) 650°C. Two multi-reaction monitoring (MRM) transitions (a quantifier and a qualifier ion for each compound) were used. The executed MRM experiments with the associated settings are summarized in Supplemental Table 2.

**Fig 2:**
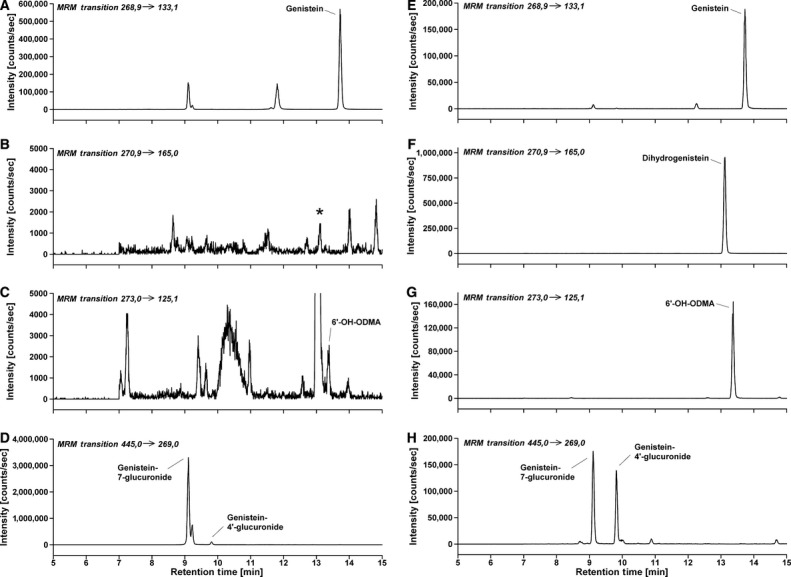
Representative MRM ion chromatograms using the respective ion transition for each metabolite. (A–D) represents the chromatograms of a plasma sample from a rat showing the amounts of the selected genistein metabolites after receiving the genistein-supplemented diet (E–H) shows chromatograms of an extracted control plasma sample spiked with 50 nmol/l of each analyte. Peak * in chromatogram B was not confirmed as dihydrogenistein with the second qualifier transition. Chromatographic and mass spectrometric conditions are described in detail in the experimental section.

Separation of the isoflavones was performed on a Phenomenex Synergi Hydro-RP column (3.0 mm internal diameter, 150 mm length, 4 μm) with an oven temperature of 40°C. Solvent A was a 0.1% (v/v) formic acid solution and solvent B was acetonitrile (VWR, LC grade). Flow rate was 0.6 ml/min., the injection volume 20 μl. The elution profile was as follows: 0–1 min. isocratic with 5% B, 1–16 min. from 5% to 50% B, 16–17 min. from 50% to 100% B, 17–20 min. isocratic with 100% B, 20–21 min. from 100% to 5% B and 21–30 min. isocratic with initial conditions. The limits of quantification (LOQ) in plasma, expressed as the amount of each metabolite on column, were as follows: 40 fmol for genistein, dihydrogenistein and 6′-OH-ODMA as well as 200 fmol for both genistein-monoglucuronides. The LOQ for each analyte in liver samples were as follows: 40 fmol for genistein and dihydrogenistein and 200 fmol for 6′-OH-ODMA and both genistein-glucuronides. We defined the limit of detection (LOD) as one third of the respective LOQ. Representative MRM ion chromatograms using the respective ion transition for each metabolite are given in Figure 2.

### Statistical analysis

Statistical analysis was performed with PASW Statistics 18 (IBM, Chicago, IL, USA). Data were analysed for normality of distribution (Kolmogorow–Smirnov and Shapiro–Wilk test) prior to Mann–Whitney *U*-test. Data are expressed as means with their standard errors and significance was accepted at *P* < 0.05.

## Results

### Cell culture experiments

#### Cytotoxicity

Resveratrol, cyanidin, malvidin, catechin, naringenin, quercetin, isorhamnetin, genistein, daidzein and G7-MGluc as well as G7-MSulf and G4′,7-DSulf did not show any cytotoxicity up to a concentration of 25 μmol/l. The highest non-toxic concentrations for naringenin and luteolin were 10 μmol/l and 5 μmol/l respectively (data not shown). These highest non-cytotoxic concentrations of the test components were then used for the subsequent reporter gene assay experiments.

#### PON1-transactivation

**Fig 3:**
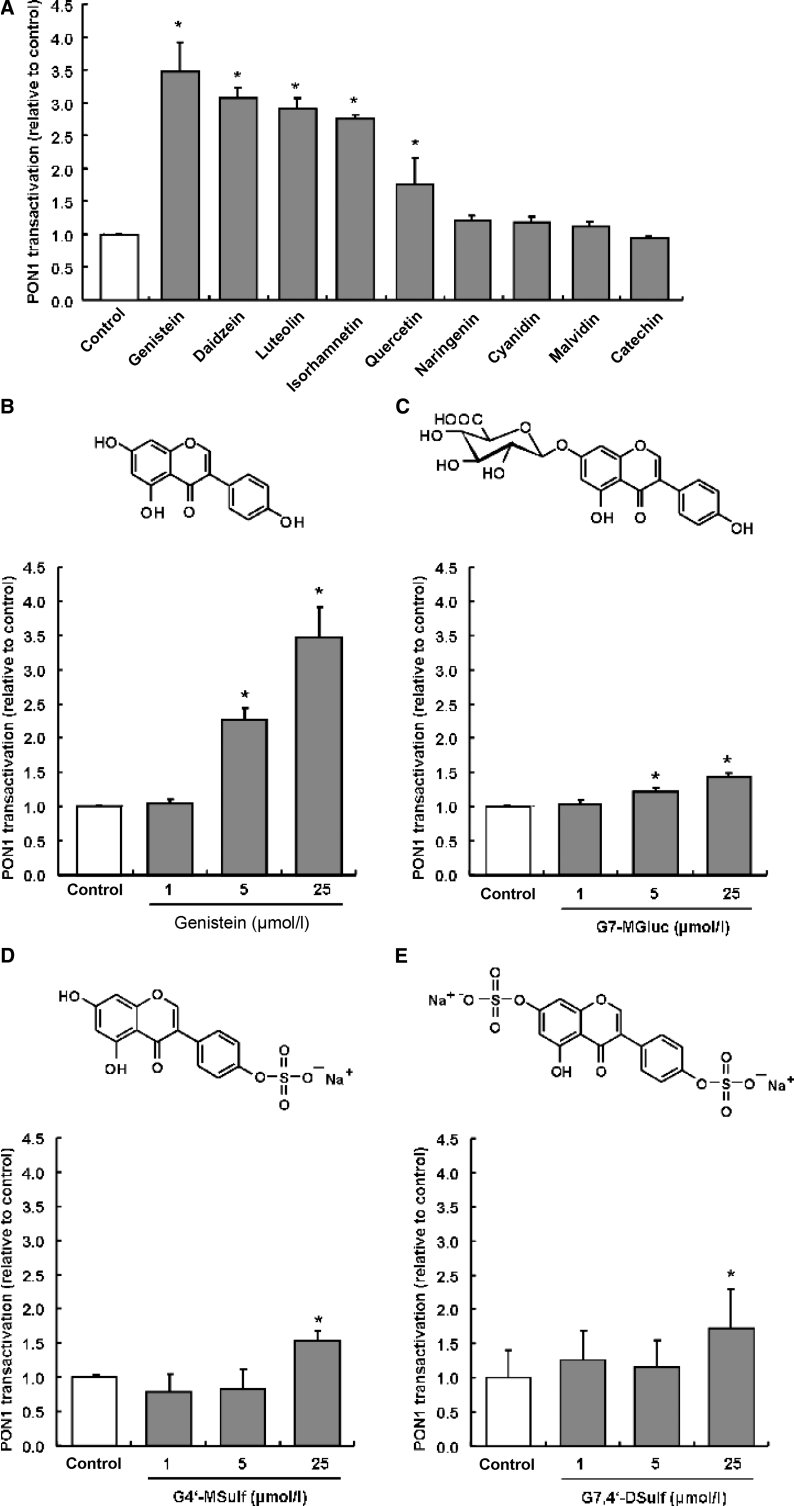
(A) Induction of PON1 transactivation by different flavonoids in stably transfected Huh7 liver cells. (B) The chemical structure of genistein and dose-dependent effect of genistein on PON1 transactivation in stably transfected Huh7 liver cells. (C) The chemical structure of the genistein-metabolite genistein-7-glucuronide (G7-MGluc) and dose-dependent effect of G7-MGluc on PON1 transactivation in stably transfected Huh7 liver cells. *statistical significant differences at *P* ≤ 0.05; anova. (D) The chemical structure of the genistein-metabolite genistein-4′-sulfate (G4′-MSulf) and dose-dependent effect of G4′-MSulf on PON1 transactivation in stably transfected Huh7 liver cells. (E) The chemical structure of the genistein metabolite genistein-7,4′-disulfate (G7,4′-DSulf) and dose-dependent effect of G7,4′-DSulf on PON1 transactivation in stably transfected Huh7 liver cells. Reporter gene data are mean with S.E.M. of three experiments performed in triplicate.

Luciferase reporter gene activity in human Huh7 liver cells, stably transfected with a human PON1 promoter fragment, was significantly induced by the treatment with genistein followed by daidzein, luteolin, isorhamnetin and quercetin. Other flavanoids such as naringenin, cyanidin, malvidin and catechin did not affect PON1 transactivation (Fig. 3A).

Genistein-induced PON1 in a dose-dependent manner as summarized in Figure 3B. Although 1 μmol/l genistein did not result in a significant induction of PON1 transactivation, the supplementation of 5 and 25 μmol/l genistein resulted in a significant (*P* < 0.05) 2.3 and 3.5-fold induction of PON1 transactivation as compared with control. In contrast, the genistein-metabolites G7-MGluc, G4′-MSulf and G7,4′-DSulf were only weak inducers of PON1 transactivation in our reporter gene assay as shown in Figure 3C,D,E.

**Fig 4:**
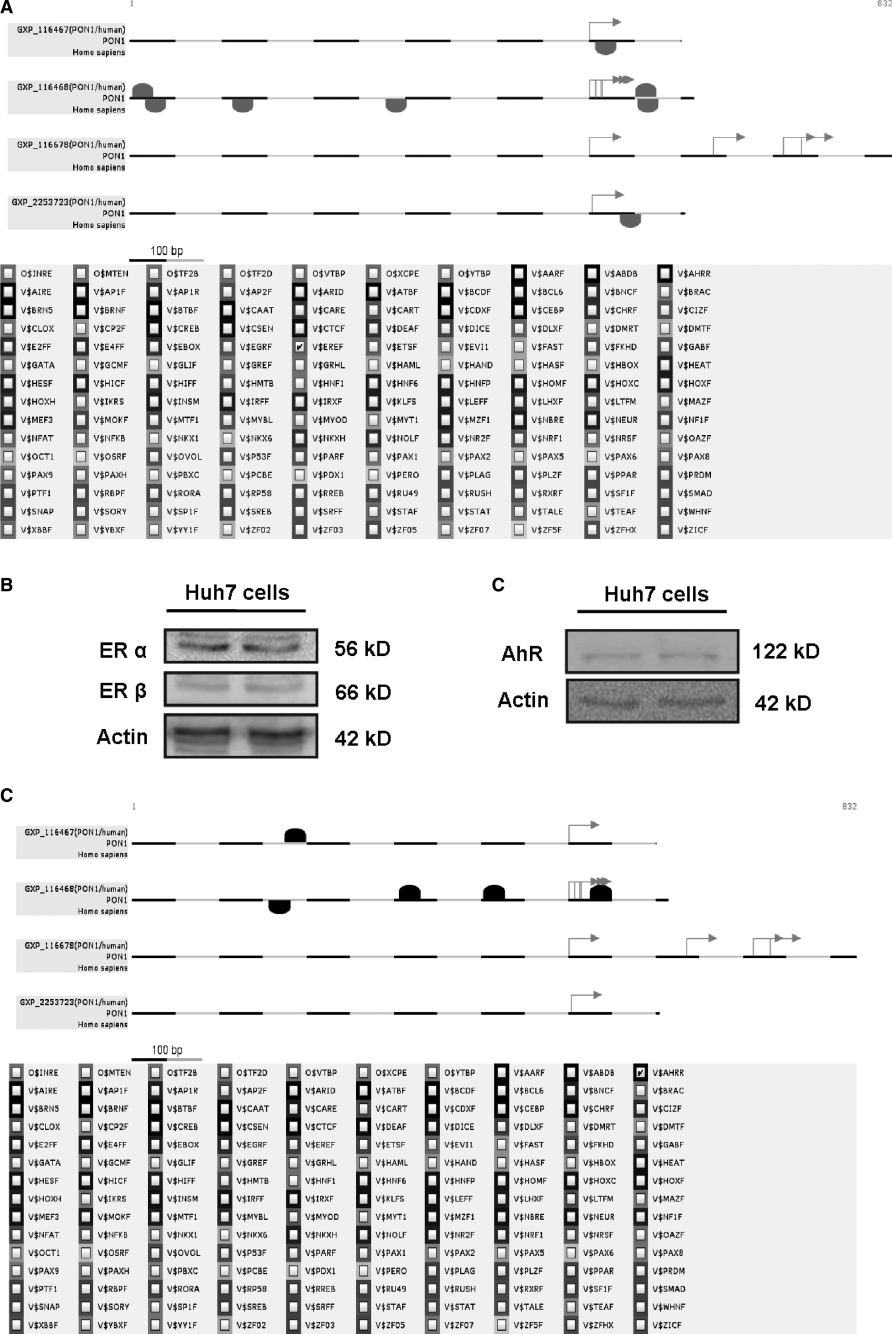
(A) Oestrogen response elements (ERE) in alternative promoter sequences of the PON1 gene. ERE binding sites are depicted in alternative promoter sequences of PON1 (GXP_116467, GXP_116464, GXP_116678, GXP_2253723) relative to transcription start site (red arrow). (B) Western blotting of oestrogen receptors α and β in Huh7 whole cell lysates indicates presence of both oestrogen-receptor variants in Huh7 cells. Actin served as loading control. (C) Western blotting of aryl hydrocarbon receptor (AhR) in Huh7 whole cell lysates indicates presence of AhR in Huh7 cells. Actin served as loading control. (D) Aryl hydrocarbon receptor-binding sites in alternative promoter sequences of the PON1 gene. ERE-binding sites are depicted in alternative promoter sequences of PON1 (GXP_116467, GXP_116464, GXP_116678, GXP_2253723) relative to transcription start site (red arrow).

We conducted a systematic *in silico* analysis to identify oestrogen response elements in the PON1 promoter. Four alternative promoter sequences have been identified for PON1 genes. In total eight binding sites for oestrogen have been found in three of the four promoter sequences of PON1 (Fig. 4A). The presence of ERα and ERβ in Huh7 cells was confirmed by Western blotting. As depicted in Figure 4B, both ER variants were found in Huh7 protein lysates.

**Fig 5:**
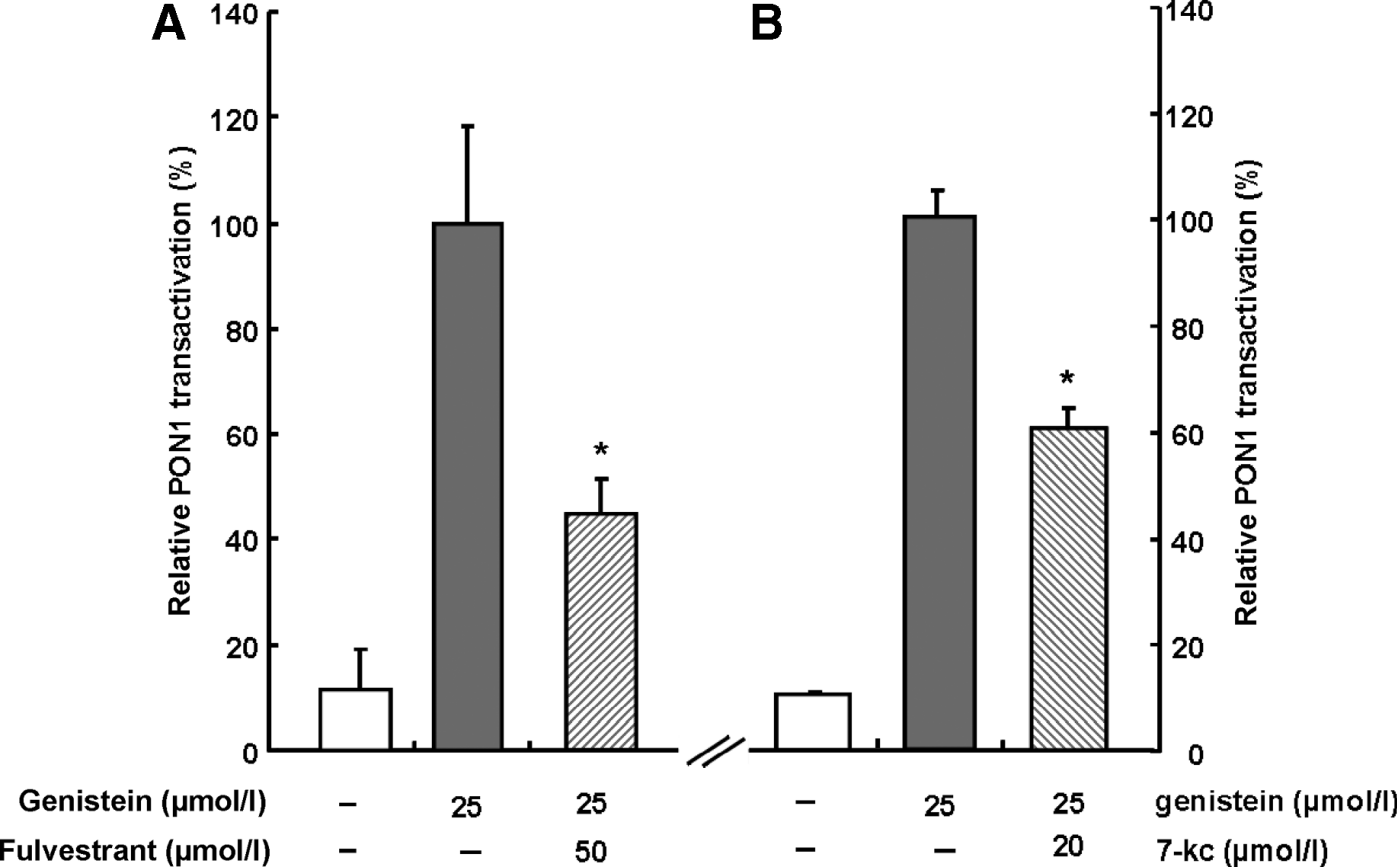
(A) Inhibition of the genistein-mediated PON1 transactivation by the oestrogen-receptor antagonist fulvestrant in stably transfected Huh7 liver cells. Cells were treated for 48 hrs. Data are mean + S.E.M. of three experiments performed in triplicates. (B) Inhibition of the genistein-mediated PON1 transactivation by the aryl hydrocarbon receptor antagonist 7-ketocholesterol (7-kc) in stably transfected Huh7 liver cells. Cells were treated for 48 hrs. Data are mean + S.E.M. of three experiments performed in triplicates. *Mean values were significantly different from the genistein treatment without 7-ketocholesterol (*P* ≤ 0.05), Mann–Whitney *U*-test.

To evaluate whether the inducing effect of genistein on PON1 transactivation is mediated *via* oestrogen receptors, we then inhibited the oestrogen-receptor using fulvestrant, an oestrogen-receptor antagonist. As shown in Figure 5, inhibition of oestrogen-receptors by fulvestrant significantly decreased genistein-mediated PON1 transactivation.

In addition to oestrogen-receptor binding sites, we found five aryl hydrocarbon receptor (AhR)-binding sites in the PON1 promoter *in silico* (Fig. 4D). The presence of AhR in Huh7 cells was again confirmed by Western blotting as shown in Figure 4C. Interestingly, genistein mediated-PON1 transactivation was decreased in the presence of 7-ketocholesterol (7-kc), an AhR antagonist (Fig. 5). We also measured the effect of fulvestrant and 7-kc only on PON1 transactivation and observed a ∼40% inhibition and 98% inhibition, respectively, of PON1 transactivation as compared with untreated controls (data not shown).

### Rat study

#### Concentration of genistein and metabolites in plasma and liver samples

The concentrations of genistein, dihydrogenistein, 6′-OH-ODMA as well as of G7-MGluc and G4′-MGluc were determined in liver samples as summarized in Table 1. Other genistein conjugates like genistein-sufates or mixed sulfoglucuronides were not determined.

**Table 1:** Concentration (mean ± SD) and concentration range of genistein and selected metabolites in plasma and liver tissue of rats supplemented with 2 g genistein/kg diet over three weeks. nd, not detected, <1.3 nM for dihydrogenistein in plasma; <7.7 nmol/kg for 6′-OH-ODMA in liver; <1.7 nmol/kg for dihydrogenistein in liver

Genistein metabolite	Rat plasma (nmol/l)	Rat liver (nmol/kg)
Genistein	92 ± 37 (44–148)	1679 ± 873 (872–3053)
Dihydrogenistein	nd	3 ± 3 (nd – 6)
6′-OH-ODMA	<4	5 ± 6 (nd – 13)
G7-MGluc	591 ± 145 (309–765)	534 ± 269 (242–1002)
G4′-MGluc	21 ± 12 (8–36)	21 ± 11 (9–35)

nd, not detected; 6′-OH-ODMA, 6′-Hydroxy-*O*-desmethylangolensin; G7-MGluc, Genistein-7-glucuronide; G4′-MGluc, Genistein-4′-glucuronide.

The average concentration of the genistein aglycone in the plasma of the genistein-supplemented rats was six times lower as compared with G7-MGluc as summarized in Table 1. Dihydrogenistein 6′-OH-ODMA as well as G4′-MGluc were present in the plasma if at all, only in trace amounts. In the liver, the average concentration of the genistein aglycone was higher than that of the G7-MGluc indicating that the portion of unconjugated genistein is much higher in tissues as compared with plasma. In control rats which did not receive genistein in their diet, the selected genistein metabolites were not detectable (<LOD; see Materials and methods Section).

#### Hepatic PON1 mRNA and protein levels, and PON1 activity in plasma

**Fig 6:**
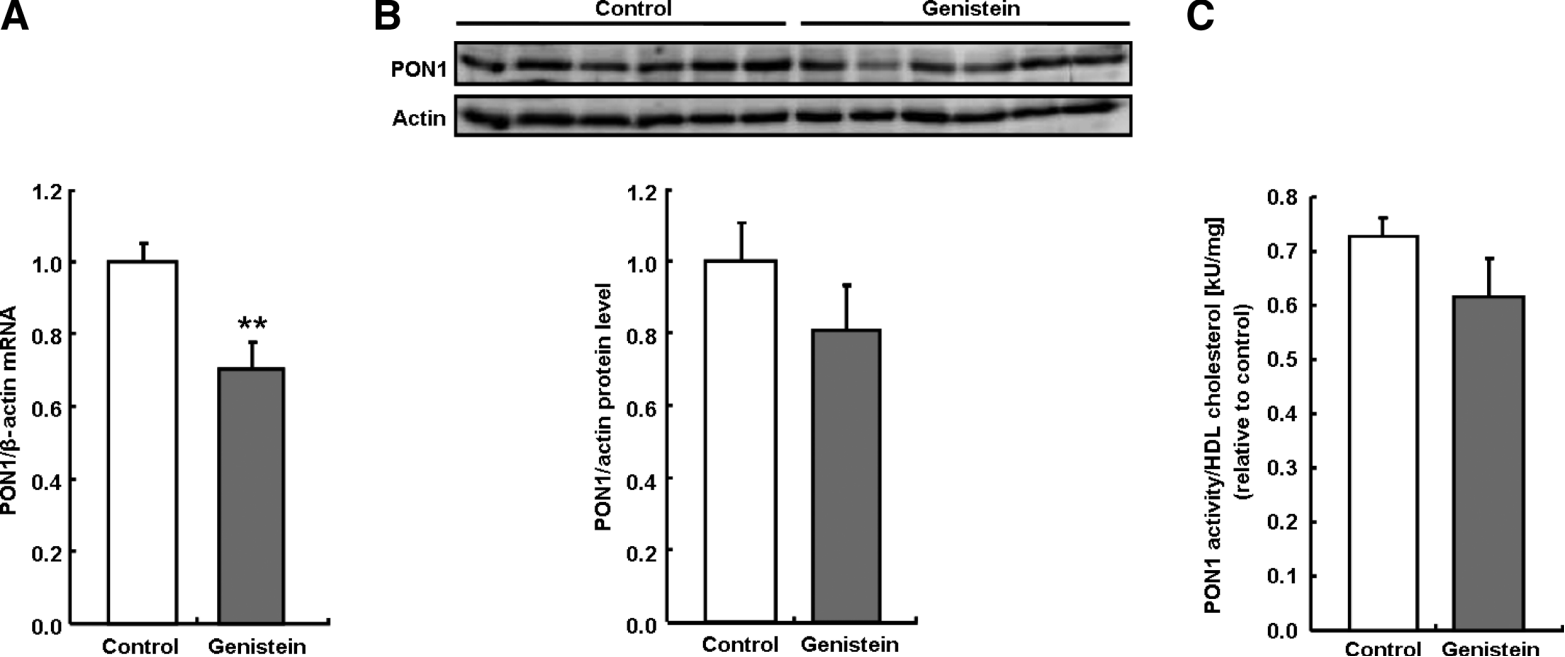
Effect of a 3-week supplementation with dietary genistein (2 g/kg) on PON1 mRNA (A) and protein levels (B) as well as plasma PON1 activity (C) in male Wistar Unilever rats compared with control-fed rats. ** indicate statistical significant differences at *P* ≤ 0.01, *t*-test.

Genistein-supplemented rats exhibited significantly lower hepatic PON1 mRNA (Fig. 6A) and slightly lower PON1 protein levels (Fig. 6B) as compared with control rats receiving the genistein-free basal diet. Furthermore, PON1 activity in plasma as well as plasma HDL (47 mg/dl *versus* 37 mg/dl) were not significantly different between control rats and rats receiving the genistein-enriched diet (Fig. 6C).

## Discussion

In our reporter gene assay, the isoflavone genistein exhibited the most potent induction of PON1-transactivation, followed by the isoflavone daidzein, the flavone luteolin and the flavonols isorhamnetin and quercetin. In contrast, the anthocyanidins malvidin and cyanidin exhibited basically no PON1-inducing activity at 25 μmol/l. Given their relatively low plasma concentration (<1 μmol/l) [40] it is unlikely that anthocyanidins may affect PON1 *in vivo*. Furthermore, catechins, known to be relatively potent free radical scavengers *in vitro* [41–43], were less potent than isoflavones in inducing PON1 transactivation. Thus, the free radical scavenging properties of flavonoids *in vitro* do not seem to be positively associated with their PON1-inducing activity. However, it has to be taken into account that flavonoids such as naringenin have been reported to inhibit PON1 activity both in human serum and when purified enzymes were used. PON1 was inhibited by naringenin in a competitive-type inhibition pattern [44]. In contrast, in our study PON1 transactivation was not significantly changed by naringenin, whereas other studies even reported an induction of PON1 transactivation and PON1 gene expression in Huh7 cells [33]. Differences between our data and data reported by the literature may be because of differences in the naringenin concentrations administered.

The underlying mechanism by which genistein induced PON1 in Huh7 cells was investigated in mechanistic cell culture studies. Previously it has shown that Huh7 cells express ERα [45]. In accordance with literature data, we found low levels of ERα and ERβ in Huh7 whole cell lysates (Fig. 6). In addition, our *in silico* promoter analysis suggest eight putative oestrogen-binding sites in the PON1 promoter. As genistein-mediated PON1 induction was inhibited by the oestrogen-receptor antagonist fulvestrant, it may be hypothesized that the induction of PON1 because of genistein could be partly be *via* an oestrogen-receptor-dependent signal transduction pathway. However, it needs to be taken into account that the fulvestrant concentration, as used in our Huh7 cell culture studies, was manifold higher than those previously reported in the literature [46–49]. Furthermore, we found putative AhR binding sites in the PON1 promoter *in silico*. 7-ketocholesterol is an AhR antagonist [33]. In the presence of 7-ketocholesterol genistein-mediated PON1 transactivation in Huh7 cells was significantly diminished suggesting that genistein may partly activate PON1 *via* an AhR-dependent mechanism. Similarly, it has shown that the flavonol quercetin acts as an AhR ligand, thereby driving PON1 gene expression [33].

Although genistein was the most potent flavonoid test compound in inducing PON1 transactivation in the reporter gene assay, 5 μmol/l genistein were necessary to induce a significant PON1 transactivation in our Huh7 cell culture model. In the present rat study, no induction of PON1 mRNA, protein and activity levels were evident in response to the dietary genistein supplementation. This seems plausible as the average concentrations of genistein and its metabolites in plasma and liver were far away from concentration of 5 μM required for PON1 induction in our reporter gene asssay. However, the effect of a long-term feeding with genistein on PON1 activity needs to be investigated in future studies.

It is well known that isoflavones are intensely metabolized in the gut and in the liver by glucuronyl transferases and sulfotransferases [50–52]. The formation of glucuronidated, sulfated and sulfoglucuronidated metabolites converts isoflavones into more water-soluble products which in turn affect their biological activity [51, 53]. Our reporter gene data demonstrate that glucuronidation and sulfatation of genistein decreases its PON1-inducing activity. Accordingly, previous data with regard to the flavanoid quercetin also suggest that glucuronidation, which masks important hydroxyl groups of the flavonoid molecule, diminishes its potency to induce PON in cultured macrophages [34]. Thus, the lack of induction of PON1 by dietary genistein, as observed in this study, may also be partly related to the fact that genistein is mainly present in rat plasma in its conjugated forms. Accordingly, the glucuronidated genistein G7-MGluc was only a poor inducer of PON1 transactivation as shown in our reporter gene assay.

Overall, present data suggest that genistein may be a PON1 inducer in cultured hepatocytes *in vitro* but not in rats *in vivo*. A mismatch between *in vitro* and *in vivo* data with regard tp PON1-inducing activity by plant bioactives has not only been observed for genistein, as reported here but also for other secondary plant metabolites including quercetin [54], curcumin [55] and allyl isothiocyanate [56]. It also clearly demonstrates that cell culture data with regard to the induction of PON1 by dietary flavonoids should always be verified in appropriate *in vivo* models before any conclusion regarding their physiological relevance is drawn.

## Supporting information

**Table S1** Average recovery rates of genistein and selected metabolites determined using the experimental protocol described in Material and methods

**Table S2** Settings used for the experiments performed in the MRM mode using a 5500 Qtrap (AB Sciex)
