# Erratum

**DOI:** 10.1111/jcmm.12127

**Published:** 2014-09-02

In [1], the fourth author's name should be Sebastian T. Soukup (first name, middle name, last name).

The authors wish to apologise for any misunderstanding or inconvenience caused.
