# Supplementary material for: Genistein as a potential inducer of the anti-atherogenic enzyme paraoxonase-1: studies in cultured hepatocytes in vitro and in rat liver in vivo
Source: J Cell Mol Med. 2012 Sep 26;16(10):2331–41. doi: 10.1111/j.1582-4934.2012.01542.x (PMC3823426; doi:10.1111/j.1582-4934.2012.01542.x)
Supplement: Supplementary file 1 [file jcmm0016-2331-SD1.doc]

**Supplemental Table 1: Average recovery rates of genistein and selected metabolites determined using the experimental protocol described in Material and Methods.**

| Analyte | Plasma | | Liver | |
| --- | --- | --- | --- | --- |
|  | Recovery [%] | Error [%] | Recovery [%] | Error [%] |
| Genistein | 93.1 | 21.3 | 91.2 | 5.2 |
| Dihydrogenistein | 100.5 | 4.1 | 80.3 | 5.3 |
| 6’-OH-ODMA | 57.6 | 7.9 | 38.6 | 2.6 |
| G4‘-MGluc | 73.0 | 5.6 | 69.0 | 2.8 |
| G7-MGluc | 77.2 | 7.2 | 64.2 | 4.9 |
| ^13^C_3_-G7-MGluc | 78.4 | 6.8 | 61.5 | 3.5 |
| ^13^C_3_-Daidzein | 105.8 | 12 | 84.5 | 4.6 |
