# Supplementary material for: Genistein as a potential inducer of the anti-atherogenic enzyme paraoxonase-1: studies in cultured hepatocytes in vitro and in rat liver in vivo
Source: J Cell Mol Med. 2012 Sep 26;16(10):2331–41. doi: 10.1111/j.1582-4934.2012.01542.x (PMC3823426; doi:10.1111/j.1582-4934.2012.01542.x)
Supplement: Supplementary file 2 [file jcmm0016-2331-SD2.doc]

**Supplemental Table 2: Settings used for the experiments performed in the MRM mode using a 5500 Qtrap (AB Sciex).**

| Compound | Transition | Mass 1 | Mass 2 | Dwell time [ms] | DP [V] | CE [V] | CXP [V] |
| --- | --- | --- | --- | --- | --- | --- | --- |
| Genistein | 1 | 268.9 | 133.1 | 20 | -160 | -42 | -11 |
|  | 2 | 268.9 | 65.0 | 20 | -160 | -70 | -6 |
| Dihydrogenistein | 1 | 270.9 | 165.0 | 20 | -135 | -29 | -13 |
|  | 2 | 270.9 | 65.1 | 20 | -135 | -68 | -5 |
| 6’-OH-ODMA | 1 | 273.0 | 125.1 | 20 | -120 | -26 | -11 |
|  | 2 | 273.0 | 229.1 | 20 | -120 | -24 | -18 |
| G4‘-MGluc | 1 | 445.0 | 269.0 | 20 | -160 | -40 | -5 |
|  | 2 | 445.0 | 113.0 | 20 | -160 | -23 | -9 |
| G7-MGluc | 1 | 445.0 | 269.0 | 20 | -140 | -41 | -5 |
|  | 2 | 445.0 | 113.0 | 20 | -140 | -22 | -9 |
| ^13^C_3_-G7-MGluc | 1 | 448.0 | 272.0 | 20 | -130 | -38 | -5 |
|  | 2 | 448.0 | 113.1 | 20 | -130 | -22 | -9 |
| ^13^C_3_-Daidzein | 1 | 256.0 | 134.1 | 20 | -165 | -54 | -11 |
|  | 2 | 256.0 | 226.1 | 20 | -165 | -38 | -18 |

Transition 1: quantifier; transition 2: qualifier; DP: declustering potential; CE: collision energy voltage; CXP: collision cell exit potential
